# ‘It is just the way it was in the past before I went to test’: a qualitative study to explore responses to HIV prevention counselling in rural Tanzania

**DOI:** 10.1186/s12889-016-3109-7

**Published:** 2016-06-08

**Authors:** Caoimhe Cawley, Alison Wringe, Joyce Wamoyi, Shelley Lees, Mark Urassa

**Affiliations:** London School of Hygiene and Tropical Medicine, Keppel Street, London, WC1E 7HT UK; National Institute for Medical Research, Mwanza, Tanzania

**Keywords:** HIV testing and counselling, HIV prevention counselling, sexual behaviour, Sexual risk reduction, HIV prevention, Tanzania

## Abstract

**Background:**

Voluntary counselling and testing (VCT) for HIV first evolved in Western settings, with one aim being to promote behaviours which lower the risk of onward transmission or acquisition of HIV. However, although quantitative studies have shown that the impact of VCT on sexual behaviour change has been limited in African settings, there is a lack of qualitative research exploring perceptions of HIV prevention counselling messages, particularly among clients testing HIV-negative. We conducted a qualitative study to explore healthcare worker, community and both HIV-negative and HIV-positive clients’ perceptions of HIV prevention counselling messages in rural Tanzania.

**Methods:**

This study was carried out within the context of an ongoing community HIV cohort study in Kisesa, northwest Tanzania. Nine group sessions incorporating participatory learning and action (PLA) activities were conducted in order to gain general community perspectives of HIV testing and counselling (HTC) services. Thirty in-depth interviews (IDIs) with HIV-negative and HIV-positive service users explored individual perceptions of HIV prevention counselling messages, while five IDIs were carried out with nurses or counsellors offering HTC in order to explore provider perspectives.

**Results:**

Two key themes revolving around socio-cultural and contextual factors emerged in understanding responses to HIV prevention counselling messages. The first included constraints to client-counsellor interactions, which were impeded as a result of difficulties discussing private sexual behaviours during counselling sessions, a hierarchical relationship between healthcare providers and clients, insufficient levels of training and support for counsellors, and client concerns about confidentiality. The second theme related to imbalanced gender-power dynamics, which constrained the extent to which women felt able to control their HIV-related risk.

**Conclusion:**

Within the broader social context of a rural African setting, HIV prevention counselling based on a Western model of individual-level agency seems unlikely to make a significant contribution to sexual behaviour change until there is greater recognition by counsellors of the ways in which power dynamics within many relationships influence behaviour change. More culturally relevant counselling strategies and messages and infrastructural improvements such as additional training for counsellors and counselling rooms which ensure privacy and confidentiality, may lead to better outcomes in terms of sexual risk reduction.

## Background

Voluntary counselling and testing (VCT) for HIV first evolved in Western settings, with a strong focus on coping and support mechanisms for those diagnosed HIV-positive, as well as on the promotion of behaviours to lower the risk of onward transmission or acquisition of HIV [1]. While there is a paucity of evidence to guide the theoretical constructs upon which HIV prevention counselling advice is expected to act, many individual-level models of behaviour change focus on aspects of individual agency such as intentions, attitudes, beliefs and feelings of self-efficacy regarding sexual risk reduction [2]. However, social and cultural norms, as well as structural factors such as poverty, are likely to be important determinants of behaviour in African and other settings where identity is strongly influenced by family, community and other social factors [3].

As Western HIV testing norms have been imported into African settings, this has been accompanied by assumptions that, as in Western settings, counselling advice can help to encourage reductions in high risk sexual behaviours such as multiple concurrent partnerships or increases in the frequency and consistency of condom use [4, 5]. The sharing of personal information by the client with the counsellor is seen as essential to the counselling process, as is the joint identification of risk behaviours and possible strategies to reduce them [1, 6]. A number of studies have highlighted some of the challenges associated with the provision of HIV risk reduction counselling in African settings, including difficulties experienced by counsellors when trying to balance the contrasting aims of VCT as a public health intervention with specific goals, but also an individually targeted counselling intervention which aims to respect client autonomy and choice [7]. Other challenges include difficulties adapting standard HIV testing guidelines to local settings [8, 9], and difficulties and discomfort experienced by counsellors when talking with clients about sexuality and sexual behaviour [10, 11]. Quantitative evidence to support the role of HIV testing and counselling (HTC) in sexual behaviour change in sub-Saharan Africa is weak, particularly among HIV negative individuals [12–14]. While a few qualitative studies have explored clients’ perspectives of and responses to HIV prevention counselling messages in African settings, many of these have focussed on HIV-positive individuals [15–17] and few have included individuals testing HIV-negative [18, 19].

In order to better understand the processes through which HTC may contribute to sexual behaviour change and HIV prevention in a rural area of northwest Tanzania, we conducted a qualitative study to explore healthcare worker, community and both HIV-negative and HIV-positive clients’ perceptions of HIV prevention counselling advice provided at VCT or at an antenatal provider-initiated testing and counselling (PITC) service (antenatal PITC has a greater focus on diagnosis and referral of HIV-positive patients compared to VCT, but still includes counselling on HIV risk reduction regardless of HIV-status [20]).

## Methods

### Study setting and design

This study was carried out within the context of a community HIV cohort study in Kisesa in rural northwest Tanzania, where regular rounds of demographic and HIV serological surveillance (sero-surveys) have been ongoing since 1994 [21]. HIV prevalence in the study area was estimated at 6.5 % in 2010, and most residents are subsistence farmers. VCT services were first provided as part of the 2003–2004 sero-survey, with further provision of VCT during three additional sero-survey rounds between 2006 and 2013. A permanent VCT clinic opened within the study area’s main health centre in 2005, while PITC has been routinely offered to pregnant women attending the health centre’s antenatal clinic (ANC) since the end of 2008. Temporary outreach VCT services are occasionally provided by governmental or non-governmental organisations. Although they have improved over time, rates of HIV testing uptake in Kisesa remain lower than the national average, with coverage of testing being approximately 40 % among men and 50 % among women in 2010 [22], compared to 47 % among men and 62 % among women in the country as a whole in 2011/2012 [23].

Qualitative data collection took place between February and April 2012. Nine group activities incorporating participatory learning and action (PLA) activities were conducted in order to gain general community perspectives of HTC services, as well as to serve as a source of recruitment for in-depth interviews (IDIs). Thirty IDIs with HIV-negative and HIV-positive service users explored individual experiences of completing HTC, while five IDIs were carried out with nurses or counsellors offering HTC in order to explore provider perspectives. The IDIs with service users and healthcare workers provided the richest source of information in terms of understanding responses to HIV prevention counselling messages, and so the majority of findings reported here are drawn from these.

### Sampling, recruitment and data collection

PLA activities were carried out with four female and five male groups of six to twelve participants, stratified by rural or urban residence and age-group (18–34 or 35–60 years). A combination of purposive sampling and snowball techniques were used. Firstly, four to six individuals were purposively selected from a sampling frame constructed from the demographic and sero-surveillance datasets, which included some ‘seeded’ individuals who were known or reported to have used HTC services, as well as some who had not used HTC. The ‘seeded’ focus group approach has previously been used in this setting [24], and allowed us to include HTC clients in the group activities without inadvertently revealing their HTC-use status during recruitment or data collection activities. Next, each purposively sampled individual was asked to invite a neighbour or friend from their village from the same age group to participate. In total 48 purposively sampled men and women were invited to participate, of whom 40 attended and 32 brought a neighbour or friend, resulting in a total of 72 participants (41 men and 31 women). The PLA activities included brainstorming activities using flip-chart paper to express ideas and opinions relating to the perceived usefulness of HTC services, positive and negative aspects of service provision, and opinions relating to counsellors.

IDIs were carried out with 15 men and 15 women who had used HTC services, 10 of whom were HIV-positive (five men and five women). The majority of interviewees (*n* = 25) were recruited via the group activities. At the end of the activity, each participant was asked privately whether s/he was willing to discuss previous use of HTC. If the participant agreed and was an HTC user, s/he was invited to participate in an IDI on experiences of going for HTC. The discussion guide included topics on motivations for going for HTC, impressions of counsellors, information and advice shared with and received from counsellors, attitudes and feelings regarding the counselling advice received and regarding sexual risk reduction, and experiences of disclosing HTC use to partners or other family members. Five HIV-positive individuals were recruited for IDIs via the HIV care and treatment clinic (CTC) at Kisesa health centre in order to recruit individuals who may and may not have accessed antiretroviral therapy.

Five IDIs were carried out with healthcare workers, purposively sampled to include individuals providing VCT (*n* = 2), PITC at the ANC (*n* = 2), and one member of the District Health Management Team who provided training, supervision and support for counsellors. Topic guides for healthcare workers providing HTC services included length of time and experience providing HTC, the content and information shared during pre and post-test counselling sessions, community perceptions of HTC, and levels of training and support received. The IDI with the HTC trainer covered issues relating to the content of counsellor training programmes, as well as ongoing professional development, support and supervision for counsellors.

All group activities and IDIs were conducted in Swahili by same gender researchers, with the exception of the IDI with the HTC trainer (a woman who was interviewed by a man). Study activities were audio-recorded following informed consent from participants, and then transcribed and translated into English.

### Data analysis

Coding and analysis of transcripts was carried out in Nvivo10, guided by a framework approach [25]. After familiarisation with the data, an initial coding frame was developed based on the research questions, but also drawing on concepts derived from theories of behaviour change. Codes were also generated inductively, grounded in the accounts of study participants. The initial coding frame developed by the lead author (CC) was reviewed by co-authors (AW, SL and JW) and revised according to emerging concepts, which were grouped under overarching themes. The refined coding-frame was then applied to all transcripts. Data were then synthesized, distilling the coding to explore the emerging patterns and meanings in terms of sexual behaviour change and HIV-prevention, and to provide explanations for findings.

## Results

The characteristics of the 30 HTC clients who completed IDIs are shown in Table 1. Most clients had received their most recent HTC at the walk-in VCT clinic at Kisesa Health Centre and had tested within the last year. In total 18 were HIV-negative, 10 were HIV-positive and two were of unknown HIV status.

**Table 1:** Characteristics of HTC users participating in IDIs

	Male (*n*=15)	Female (*n*=15)
Age group		
18–24	4 (26.7 %)	3 (20.0 %)
25-34	2 (13.3 %)	4 (26.7 %)
35–44	4 (26.7 %)	4 (26.7 %)
>45	5 (33.3 %)	4 (26.7 %)
Location of most recent HTC		
Community outreach-VCT (during sero-survey)	1 (6.7 %)	
Walk-in VCT clinic (Kisesa health centre)	8 (53.3 %)	9 (60.0 %)
Other VCT^a^	4 (26.7 %)	3 (20.0 %)
Antenatal PITC		3 (20.0 %)
Unknown	2 (13.3 %)	
Time since most recent HTC		
<1 year	9 (60.0 %)	7 (46.7 %)
2–4 years	4 (26.7 %)	3 (20.0 %)
>4 years		4 (26.7 %)
Unknown	2 (13.3 %)	1 (6.7 %)
HIV Status		
Negative	9 (60.0 %)	9 (6.00 %)
Positive	5 (33.3 %)	5 (33.3 %)
Unknown	1 (6.7 %)	1 (6.7 %)
Area of residence		
Rural	7 (46.7 %)	9 (60.0 %)
Trading centre (urban)	8 (53.3 %)	6 (40.0 %)
Level of education		
None	1 (6.7 %)	
Primary	7 (46.7 %)	13 (86.7 %)
Secondary or higher	7 (46.7 %)	2 (13.3 %)
Unknown		
Marital status		
Married monogamous	7 (46.7 %)	9 (60.0 %)
Married polygamous	1 (6.7 %)	
Separated/divorced	2 (13.3 %)	2 (13.3 %)
Widowed	2 (13.3 %)	3 (20.0 %)
Single (never married)	3 (20.0 %)	1 (6.7 %)

^a^Other outreach VCT service within Kisesa, or VCT service outside Kisesa ward

Two key themes emerged in understanding responses to HIV prevention counselling messages in Kisesa. These revolved around socio-cultural and structural factors, including i) constraints to client-counsellor interactions, and ii) gender norms and relationship dynamics. We found only moderate support for the idea that individual attributes such as intentions or feelings of self-efficacy had an impact on behaviour change following HTC, as perceptions of HIV counselling advice were affected by the broader socio-cultural and contextual factors that shaped participants’ lives. Findings are presented below in each of the two key thematic areas.

### Constraints to client-counsellor interactions

#### Difficulties talking about sexual behaviour

Difficulties in discussing sexuality were frequently reported by IDI participants, with apparent barriers in terms of clients sharing details of their private sexual lives with counsellors. This appeared to be particularly so for women, few of whom seemed to engage fully in counselling sessions by sharing detailed personal information with counsellors, or by seeking advice on specific issues. Some woman appeared unaware of the right to pro-actively seek information or advice, mentioning that counsellors should be the main speakers during the conversation. Women also appeared hesitant to ask questions or share information as a result of the sensitive nature of the subject matter.

*I didn’t ask her questions, she is the one who asked me if I have a husband. I didn’t have any question to ask her. I wondered what kind of question I could ask, I didn’t want to ask. (HIV-positive female, 45–60 years).*

A number of women reported having met with a male counsellor, and this appeared to make discussion of private sexual matters particularly difficult.

*Interviewer: What did you talk to him [the counsellor] about?Respondent: It is him who was asking as I answer…Actually I never thought in my mind to ask him any question… in general it was hard to tell a man. We could talk about other things but for…mmm it is difficult. It is just fear to tell the male counsellor, because all men are… they are of the same calibre. (HIV-negative female, 18–29 years).*

Compared to women, men more often reported feeling at ease during counselling sessions and more commonly mentioned that they had free and open discussions with counsellors.

*I felt free to talk to him as a result of the way he also talked to me. Then at once the fear I had went away and I was able to tell him about my problem… you have to explain to him so that he can be able to have a clear picture about your issues…” (HIV-negative male, 18–29 years).*

Some men appeared to feel at ease even if they met with a counsellor of the opposite sex. However, one man admitted that he withheld information from a female counsellor because he appeared to have been embarrassed or ashamed about an extra-marital affair he had had. This limited the potential contribution of counselling in terms of identifying possible strategies or plans for behavioural change.

*I never wanted to be transparent because she was an expert…she tried to interrogate me why I had gone there, I told her that I just felt like coming to test to check on my health, even though I knew I had an extra marital affair one day. I never told her about that. (HIV-negative male, 30–44 years).*

#### Hierarchical relationship between clients and counsellors

Counsellors recounted their efforts to engage clients in open discussions about their problems, and reported being dedicated to their profession. However, counsellors also reported that they felt a responsibility to help reduce the spread of HIV and to educate their clients, and were sometimes authoritative and directive in their counselling, creating a hierarchical relationship between healthcare provider and ‘patient’.

*You have to ensure that somebody has understood…. It is until he repeats everything that you have said…. and then if you realize that it is correct, then he would have understood…. You continue to advise him that it is supposed to be like this and this (Healthcare worker number 3).*

Clients reported that counsellors were kind, caring and knowledgeable, but also referred to them as ‘experts’, and stated their intentions to comply with the ‘rules’ or ‘instructions’ which they received. Counsellors’ roles as experts seemed to place them in a position of authority and respect, and this is likely to have influenced clients’ perceived abilities to ask questions or share information freely during counselling sessions.

*What I know about the guiding and counselling, it is somewhere we go so as to be given instructions. (Male group activity participant, age-group 35–60, rural residence).*

*I told her that I will receive them [the test results] despite what they will be, if it is HIV positive then you will tell me the right procedure to follow. (HIV-negative male, 18–29 years).*

Counsellors occasionally highlighted difficulties interacting with clients or engaging them in two-way discussions. One counsellor mentioned that clients were ‘always open’, although she later described the case of a woman who ‘*was not able to put it open that she had intercourse contrary to the way it was supposed to be done’ (Healthcare worker number 1)*, implying a certain moral overtone or judgement of her client’s behaviour which may have discouraged further discussion.

#### Confidentiality

Concerns about confidentiality may affect client willingness to share personal information during HTC. Most study participants reported that they felt counsellors were trustworthy and were unlikely to disclose test results or confidential information to others. However, a small number of participants reported concerns about counsellor confidentiality, including one HIV-positive woman who explained how a family member was informed about her HIV-positive status before she had received the result herself. During one group activity, male participants referred to rumours that some counsellors did not respect privacy and ‘gossiped’ about patients.

A few participants stated that a client’s HIV status could sometimes be deduced by other clients in the waiting room if an individual spent a long time with the counsellor (assumed to be HIV-positive). This may make clients reluctant to engage in lengthy counselling sessions for fear others might suspect them of being HIV-positive. During PLAs, several participants raised concerns that the counselling rooms at the health centre were not sufficiently private and that conversations between counsellors and clients might be overheard by other patients or staff. This inhibited communication and was particularly a problem at the ANC, where HTC sessions were conducted in an only partially private area.

*If the nurse and the patient talk loudly, the other people might hear. But if we had a room that was covered like this one, you could talk to the mother inside here very well. (Healthcare worker number 2).*

#### Training and support for counsellors

Healthcare workers were generally appreciative of the training they had received. However they reported that training courses were too short, and that they had not received any refresher training sessions or supervision, but that these would be useful in order to help them update and renew their skills. One nurse working at the ANC reported that she had started providing PITC before receiving any official training and that this had made her work difficult, particularly because she was unsure about how to deliver HIV-positive test results to clients.

*That time was not enough… There were many things to be learnt in two weeks… So they should have made it to be one month. It will have been better because we could learn slowly. And put all the things straight. Or the refresher courses should be there, so that people can be reminded. (Healthcare worker number 3).*

Many clients reported that they had received standardised ‘ABC’ messages from counsellors (abstain from sex, be faithful to one partner and/or use condoms), but there was generally little evidence of individual tailoring of counselling messages to clients’ particular circumstances. There also appeared to be limitations to the depth of the discussions held during counselling sessions. This is highlighted by the case of one young woman who, in response to a recommendation to use condoms, reported that she felt her partner would not be willing to do so. The counselling recommendation was to deny her partner sex, but did not seem to include a broader discussion of potential strategies to attempt to engage her partner in a dialogue on the topic.

*He told me to use the condom…. I asked him what if he refuses, if he refuses to use the condom. “Don’t have sexual intercourse with him so that he can come and test”. (HIV-negative woman 18–29 years).*

### Gender norms and relationship dynamics

Men commonly reported being satisfied with the counselling advice they had received, and seldom seemed to anticipate difficulties adhering to it. In contrast, women often highlighted concerns about revealing their attendance at HTC to their partners or about trying to discuss the advice received, reporting fears that their partners would not be willing to engage in the conversation or that they would be unhappy about certain aspects of the advice (for example recommendations to use condoms). Men and women’s perceptions of the counselling advice they received highlighted inequitable gender roles, with men feeling empowered after having gone for HTC, but women often feeling anxious or indifferent about their ability to act upon counselling advice.

*I actually felt happy because he advised me on important issues, they got in to my mind, and I accepted it that actually this advice was important to me to use it. I really felt happy. (HIV-negative male, 18–29 years).*

*You might get home and explain to your partner. When he doesn’t listen it becomes difficult. Even if I start to advise him by telling him that, please partner it is like this and this, he doesn’t want to understand. (HIV-negative woman, 30–44 years).*

*He [the counsellor] asked if I have ever used the condom, then I replied that I hadn’t…. There was not enough elaboration because some of us can’t use protection. …. Some men don’t want to use the condoms. (HIV-negative female, 18–29 years).*

Suspicions of partner infidelity were common, and many participants reported feeling at risk of HIV as a result of their partner’s rather than their own behaviour. In particular, women reported that it was expected or accepted that men could have multiple partners, and seemed to imply there was a certain inevitability to their partners’ infidelity, but that there was little they could do to change this.

*You should get home and start telling your partner. He listens to you during that time but after some time he forgets about it. He starts doing the same thing [having extra-marital partners]. (HIV-negative woman 30–44 years).*

Several women implied that it was the testing aspect of the service, rather than the counselling offered, which was most useful. One young woman described that not much had changed in her relationship after going for HTC, despite the fact that she suspected her husband of being unfaithful, but that she planned to test again the next month because she found the testing process reassuring as a way to check up on her HIV status.

*It is just the way it was in the past before I went to test… I just encouraged myself for the next month to come to go and test to be able to check my health status so as to continue protecting myself. (HIV-negative woman 18–29 years).*

Some participants reported having gone for HTC together with their partner, either because they were planning to get married or because they were pregnant, while a small number reported having gone to test together simply to confirm each other’s HIV status. A number of participants reported that couples testing would be useful to do, either because it might facilitate better commitment to sexual risk reduction by both members of the couple, and/or because it would help couples to learn about each other’s HIV status.

*After receiving all that guiding and counselling I just felt good, but there is one thing that I felt was not good, it is that I didn’t come along with my wife. I felt that it would have been better if we would have been together with my wife. (HIV-negative male, 45–60 years).*

*Maybe that is where we get enlightened and be able to say that, we are both mobilised. It is not that I get motivated alone. (HIV-negative female 30–44 years).*

## Discussion

Our study highlighted several barriers, influenced by social and contextual factors, which seemed to limit plans or intentions regarding sexual behaviour change following HTC, including limited communication between clients and counsellors during counselling sessions, and inequitable gender-power relations.

Previous studies in sub-Saharan Africa have highlighted counsellors’ difficulties talking about sexuality or intimate matters during HTC, for example because the topic was embarrassing or difficult to talk about [10, 11], and it is likely that clients share similar concerns. In some African cultures there are prescribed social norms about who should be consulted for advice regarding sexual matters, and involving an outsider in family problems may go against traditional practice [26]. Such findings may help to explain why candid discussions between clients and counsellors appeared difficult for many participants in our study, and indicate that counselling approaches which include techniques to put clients and counsellors at ease when talking about sexuality would be useful.

Previous qualitative research in Tanzania and elsewhere in sub-Saharan Africa has noted the dominant role of men within relationships [18, 27], and that men’s sexual respectability is often linked to greater sexual activity and the conquest of multiple partners, in contrast to women’s sexual respectability which is characterised by obedience, faithfulness and subservience to male partners [28, 29]. One study in Tanzania reported that men may exaggerate their numbers of sexual partners, while women were more self-effacing and tended to under-report non-marital partnerships [30]. These findings may help to explain why men in our study appeared more at ease discussing their sexual behaviour during HTC compared to women, and why men felt more empowered to act upon counselling advice.

A hierarchical relationship between healthcare provider and ‘patient’ was seen to inhibit communication between both male and female clients and counsellors during HTC, and other studies in sub-Saharan Africa have noted similar hierarchical relationships [9, 31, 32]. Lie et al. [33] note that HIV counselling guidelines tend to be prescriptive and directive by endorsing preferred behaviours (e.g. using condoms, remaining faithful to one partner), in contrast to more traditional person-centred counselling approaches which have a greater focus on client autonomy. As also observed in this study, counsellors seem to face challenges when trying to balance the contrasting aims of HTC as both a public health intervention with specific goals (i.e. to encourage sexual risk reduction), but one that is simultaneously meant to respect individual clients’ choices [7]. Training courses and HIV counselling guidelines which place greater emphasis on maintaining a neutral stance, and on techniques for engendering more equitable client-counsellor interactions, may help to improve the outcome of HTC in terms of sexual risk reduction. For example, one study in South Africa noted that counsellors who attempted to engage clients by ‘making appeals’ rather than ‘prescribing rules for living’ seemed to be more successful in gaining clients’ engagement and commitment to sexual risk reduction [7].

Research has suggested that in sub-Saharan Africa, family and community are more central to the construction of health and well-being than the individual [3, 9]. In support of this, one study reported that HIV-positive Ugandan men and women’s main motivations in preventing HIV transmission were altruistic wishes to protect sexual partners and to avoid orphaning children [34]. In African settings, HIV training modules and counselling messages which focus on broader culturally relevant altruistic messages relating to partners, children and the wider community, for example emphasising the benefits of protecting unborn children from HIV, or of maintaining the integrity and health of the family unit, may be more successful than ones which focus solely on individual interest [34].

Although studies have reported that counsellors sometimes struggle to balance confidentiality requirements with what they may perceive as being in the best interest of the patient or the community at large (for example, by disclosing an HIV-positive individual’s status to a partner or other family members without the patient’s consent, in order that the partner may protect himself or herself from HIV, or that the family may provide care and support for the patient) [8, 35], other studies have shown that confidentiality is of paramount importance for clients [9, 33]. Although most participants in our study reported that they felt counsellors were trustworthy, even small fears relating to breaches of confidentiality may have a disproportionate impact upon clients’ willingness to share sensitive information. Thus counsellor training modules should cover the importance of maintaining client confidentiality in sufficient detail, while clinic buildings and infrastructure should be sufficiently private so as to put clients (and counsellors) at ease.

As highlighted by counsellors in this study, additional aspects of counsellor training which require attention include the depth in which topics are covered, and the availability of refresher training courses and supervision and support for counsellors. Furthermore, it was also apparent that the risk reduction strategies proposed by counsellors were often limited to only include condom use or abstention, and tailored discussions that covered a fuller range of available, relevant HIV prevention tools (e.g. HIV testing for partners, treatment for partners who test positive, testing and treatment for sexually transmitted infections, and eventually, pre-exposure prophylaxis) which might represent more realistic prevention methods for some women, should also be covered in counsellors’ training courses.

A number of clients reported finding the process of learning their HIV status reassuring in itself, regardless of counselling messages received. However, this knowledge alone seemed unlikely to lead to reductions in sexual risk behaviour. Several participants reported the desire to know their HIV status as their main motivation for going for HTC, and although counselling sessions were not observed as part of this study, it is possible that a strong focus of the counselling advice was on the different possible HIV test results and how they should be interpreted, rather than on sexual risk reduction. Previous studies have suggested that ‘de-linking’ testing and counselling service provision might help clients to focus on counselling messages and thus more effectively support behaviour change [19, 36], but this hypothesis remains untested.

A relatively small number of clients reported having gone for testing together with their partner, however some expressed a desire to do so. The few individuals who reported having gone for couples-HTC reported positive experiences, including that couples might get ‘motivated’ together. Although previous studies have shown that couples-counselling is challenging for counsellors, particularly in the case of sero-discordant results [37, 38], others have reported the approach to be beneficial because discussions between husband and wife can be mediated by a third party, and because the process can help to renew commitment to the relationship by both members of the couple [39]. Much of the focus of VCT in sub-Saharan Africa to date has been on the provision of an individual service, however couples-counselling likely represents a promising option in terms of increasing the uptake of HTC services as well as helping to encourage behavioural change [40].

A limitation of this study was that HTC users may have been reluctant to share details of what was discussed with counsellors with study researchers, due to the sensitive nature of the subject matter, and due a similar hierarchical relationship between participants and researchers to that observed between clients and counsellors. In order to minimise this bias, interviewing techniques to build rapport and trust with participants were addressed during fieldworker training. In order to maximise confidentiality, all IDIs were conducted either in private rooms near Kisesa Health Centre, or in the case of rural residents, in private locations near rural dispensaries. For confidentiality reasons, individual counselling sessions were not observed as part of this study. Had we been able to do so, we might have gained more a more in-depth understanding of the nature of the interactions between clients and counsellors during counselling sessions, and of the topics covered.

## Conclusion

Within the broader socio-cultural context of a rural African setting such as Kisesa, HIV prevention counselling alone based on a Western model of individual-level agency is unlikely to make a significant contribution to sexual behaviour change. More culturally relevant counselling strategies and messages, such as those which aim to put clients and counsellors at ease when discussing sexuality or which emphasise altruistic wishes to reduce the spread of HIV, and infrastructural improvements such as additional training for counsellors and counselling rooms which ensure privacy and confidentiality, may lead to better outcomes in terms of sexual risk reduction. In the long-term, structural interventions such as improved access to education might help to reduce gender-imbalanced power dynamics and to improve outcomes among women.

## Abbreviations

ANC: antenatal clinic
CTC: care and treatment clinic
HTC: HIV testing and counselling
IDI: in-depth interview
PITC: provider-initiated testing and counselling
PLA: participatory learning and action activities
VCT: voluntary counselling and testing
